# Demethylation alters transcriptome profiling of buds and leaves in ‘Kyoho’ grape

**DOI:** 10.1186/s12870-020-02754-0

**Published:** 2020-12-04

**Authors:** Haoran Jia, Zibo Zhang, Ehsan Sadeghnezhad, Qianqian Pang, Shangyun Li, Tariq Pervaiz, Ziwen Su, Tianyu Dong, Jinggui Fang, Haifeng Jia

**Affiliations:** 1 Key Laboratory of Genetics and Fruit Development, College of Horticultural, Nanjing Agricultural University, Nanjing, China; 2 China Wine Industry Technology Institute, Yinchuan, China

**Keywords:** *DNA methylation*, *Grape*, *RNA-Seq*, 5-azacytidine, *Transcriptome*

## Abstract

**Background:**

Grape buds and leaves are directly associated with the physiology and metabolic activities of the plant, which is monitored by epigenetic modifications induced by environment and endogenous factors. Methylation is one of the epigenetic regulators that could be involved in DNA levels and affect gene expression in response to stimuli. Therefore, changes of gene expression profile in leaves and bud through inhibitors of DNA methylation provide a deep understanding of epigenetic effects in regulatory networks.

**Results:**

In this study, we carried out a transcriptome analysis of ‘Kyoho’ buds and leaves under 5-azacytidine (5-azaC) exposure and screened a large number of differentially expressed genes (DEGs). GO and KEGG annotations showed that they are mainly involved in photosynthesis, flavonoid synthesis, glutathione metabolism, and other metabolic processes. Functional enrichment analysis also provided a holistic perspective on the transcriptome profile when 5-azaC bound to methyltransferase and induced demethylation. Enrichment analysis of transcription factors (TFs) also showed that the MYB, C2H2, and bHLH families are involved in the regulation of responsive genes under epigenetic changes. Furthermore, hormone-related genes have also undergone significant changes, especially gibberellin (GA) and abscisic acid (ABA)-related genes that responded to bud germination. We also used protein-protein interaction network to determine hub proteins in response to demethylation.

**Conclusions:**

These findings provide new insights into the establishment of molecular regulatory networks according to how methylation as an epigenetic modification alters transcriptome patterns in bud and leaves of grape.

## Background

Grape (*Vitis vinifera L.*) is one of the oldest fruits among other crops and most widely cultivated all over the world. It is a commercial fruit with high nutrient value, abundant in vitamins, carbohydrates, minerals, and anthocyanin. Grape is a typical non-climacteric fruit and its growth is regulated by endogenous and external factors [1]. According to previous reports, endogenous hormones such as gibberellin (GA), ethylene (ETH), abscisic acid (ABA), and jasmonic acid (JA) have a pivotal role in the entire life cycle of grapes [2–5]. In addition, a variety of growth regulators, such as Indole-3-Butytric acid (IBA), forchlorfenuron N-(2-chloro-4-pyridyl) -N-phenylurea (CPPU), and thidiazuron (TDZ) affected various vital cellular activities [6]. Moreover, some compounds like sucrose [7], and some enzymes and transcription factors [8] are involved in grape growth and development.

Due to the fast track sequencing and high accuracy of performance, the high-throughput RNA-Seq technology is applied to various life sciences research. It can simultaneously discover and quantify transcripts [9], becoming one of the most powerful tools in quantification of global transcriptomes. RNA-Seq provides new ideas and methods for the development of genomics. In recent studies, RNA-Seq has been applied to discover the new aspects of research on various fruits such as apple [10], papaya [11], and strawberry [12] through transcriptome information, genetic resource mining, gene differential expression analysis, and alternative splicing analysis. Previously, there have been many reports on RNA-Seq technology for grape ripening regulation, metabolic process, disease resistance, and bud dormancy [13–15].

DNA methylation is a heritable epigenetic mark, which has a regulatory effect on various vital cellular activities of plants. The occurrence of DNA methylation relies on the catalysis of DNA methylation transferase (DN-MT). S-adenosyl methionine (SAM) is used as a substrate in transferring the methyl group to the 5th carbon atom of the cytosine ring in the DNA molecule to form 5-methylcytosine (5-mC). Generally, the level of DNA methylation remains stable in plants, although it depends on the methylation and demethylation balance [16–18]. Inhibitors of DNA methyltransferase like 5-azaC (5-azacytidine) reduce the level of genomic DNA methylation by blocking the DNA methylation process. There is a report that demonstrated the level of methylation of few specific genes was detected during the ripening process of tomato fruits and it was found that the level of methylation gradually decreased as maturity deepens [19]. In another report, using 5-azaC reduced the level of genome methylation and delayed fruit ripening in sweet orange [20]. Evaluation of DNA methylation status using Bisulfite Sequencing PCR (BSP) also demonstrated that demethylation induced more alternative splicing events and led to the abnormal translation process, although some genes could not be expressed normally [21]. At the same time, there is a need to discover more about the effect of 5-azaC on the methylation level changes during the metabolic growth process, mechanism of action, and regulatory networks. In the current study, we investigated the effect of methylation on ‘Kyoho’ grapes to discover the mode of action of methylation in the transcriptome level. We conducted RNA-Seq analysis on the leaves and buds of ‘Kyoho’ treated with 5-azaC and performed a cluster analysis to find key genes and pathways. In addition, the construction of interactive protein networks helps us better understand the changes in growth and development through the determination of hub proteins when demethylation occurs in grape. This study provides a more theoretical basis and molecular insights according to the effects of DNA methylation on grapes.

## Results

### Transcriptome profiling in response to demethylation

**Fig. 1:**
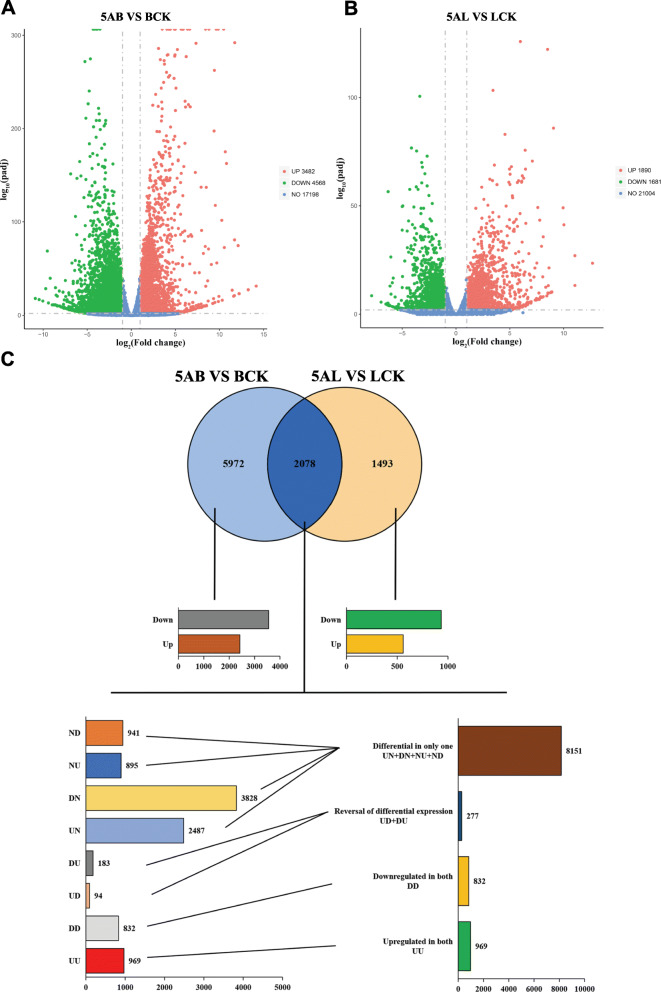
Integration analysis of gene expression in leaves and buds of ‘Kyoho’ grape after 5-azaC treatment. **a** The expression profile of DEGs in buds and (**b**) leaves. Green dots represent significantly down-regulated DEGs, and red dots represent significantly up-regulated DEGs. FDR ≤ 0.05 and |log2(Fold Change)| > 1 were defined as statistical significance. Fold change represents the change in value of FPKM between 5-azaC treatment group and control group. **c** The number of DEGs between buds and leaves after 5-azaC treatment. The overlapping DEGs of buds and leaves are shown in Venn diagram. The histogram shows the different number of DEGs including upregulated in both [UU], downregulated in both [DD], reversal of expression from bud to leaf [DU and UD] and differential in only one (either bud or leaf) [UN, DN, ND, NU]. U = upregulated, D = downregulated, and N=Neutral. 5AL; 5-azaC treated leaves, LCK; leaves control, 5AB; 5-azaC treated buds, BCK; buds control

Transcriptome sequencing of grape leaves (5-azaC treated leaves (5AL) and leaves control (LCK)) and buds (5-azaC treated buds (5AB) and buds control (BCK)) was performed after 5-azaC treatment and compared to control. We observed that the average number of total raw reads was 48.46 and 49.53 million for 5AB and BCK in buds respectively, while it was 47.41 and 47.09 million for 5AL and LCK in leaves, respectively. After removing adapter sequences and reads with low quality, total clean reads accounted for 97.54, 94.83, 95.99, and 94.12% of 5AB, BCK, 5AL, and LCK respectively. By HISAT (Hierarchical Indexing for Spliced Alignment of Transcripts), the sequencing results were mapped to the reference genome (Reference genome version: GCF_000003745.3_12X) and a higher percentage of sequences located in the reference genome (Table 1). Based on FPKM, the expression abundance of bud and leaf genes was calculated before and after of 5-azaC treatment. For buds, we found a total of 8050 DEGs, of which 3482 and 4568 genes belonged to up-regulated and down-regulated genes (Table S1, Fig. 1a). We also observed the significant differences in the expression levels of 3571 genes, including 1890 up-regulated genes and 1681 down-regulated genes when leaves treated with 5-azaC and compared to control (Table S1, Fig. 1b). In addition, the specific and non-specific DEGs in leaves and buds were represented by the VENN diagrams (Fig. 1c). To describe the common DEGs between grape leaves and buds in detail, we drew interrelated histograms to represent the different types of DEGs and reflect the differential effects of 5-azaC on different tissues of grapes.

**Table 1:** Number of sequenced reads that mapped to the grapevine genome

Sample	Total Raw Reads (M)	Total Clean Reads (M)	Total Clean Bases (Gb)	Clean^*a^ Reads Q20(%)	Clean^*b^ Reads Q30(%)	Clean Reads Ratio(%)	Total Mapping(%)	Uniquely Mapping(%)	GC_Content (%)
BCK1	47.44	45.88	6.88	98.23	94.46	96.71	90.93	88.66	45.81
BCK2	50.63	49.59	7.44	98.23	94.43	97.94	91.01	88.77	45.82
BCK3	47.32	46.36	6.95	98.37	94.82	97.97	90.95	88.64	45.98
5AB1	47.72	45.12	6.77	98.30	94.61	94.55	85.46	82.85	44.15
5AB2	53.28	50.75	7.61	98.30	94.66	95.25	86.26	83.62	44.61
5AB3	47.69	45.15	6.77	98.38	94.78	94.68	86.57	83.89	44.67
LCK1	46.23	43.81	6.57	98.42	94.90	95.17	93.40	90.95	46.24
LCK2	50.23	47.97	7.22	97.47	92.98	95.50	92.06	89.71	46.54
LCK3	45.77	44.55	6.68	98.44	94.97	97.32	93.55	91.09	46.56
5AL1	46.91	44.41	6.66	98.49	95.05	94.65	92.38	89.81	45.95
5AL2	47.43	44.68	6.72	98.40	94.83	94.19	89.83	87.28	45.02
5AL3	46.94	43.91	6.58	98.49	95.07	93.52	89.56	87.06	45.48

^a, b^Q20 and Q30 indicate the ratio of the number of bases in the filtered reads greater than 20, 30 to the total number of bases, reflecting the quality of the sequencing data

**Fig. 2:**
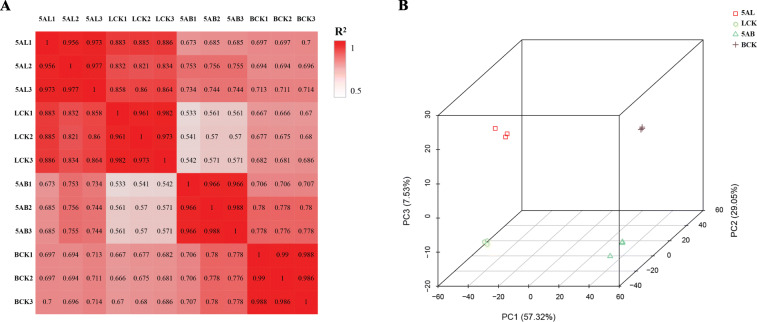
Correlation analysis and Principle component analysis (PCA) in leaves and buds of ‘Kyoho’ grape after 5-azaC treatment. **a** Correlation matrix heat map was drawn according to the Pearson correlation coefficient. The correlation strength of the sample indicates by the shade of red. Each sample includes three biological replicates. **b** Principle component analysis (PCA) of ‘Kyoho’ buds and leaves after 5-azaC treatment and compared to control. We calculated the correlation coefficient of samples within and between groups based on the FPKM values of all genes in each sample. Each sample is represented by a different icon. 5AL; 5-azaC treated leaves, LCK; leaves control, 5AB; 5-azaC treated buds, BCK, buds control

To evaluate the reliability of the experimental results, the Pearson correlation coefficient of the gene expression level was calculated for each sample and visualized in the correlation matrix heat map (Fig. 2a). DEGs expression clustering heat map was shown in Fig. S1. In addition, the principal component analysis (PCA) based on the gene expression of all treated samples revealed the segregation of dataset belonged to grape buds and leaves. PC1, PC2, and PC3 were 57.32, 29.05, and 7.53% respectively, and accounted for 93.9% of the main components (Fig. 2b). According to PCA results, we observed significant differences regarding gene expression in buds and leaves of the grapevine. Therefore, our results demonstrated that there was big discrimination between treatments and tissues but not among replicates, indicating the used samples are reasonable and reliable for subsequent experimental analysis.

### Annotations and functional analysis of DEGs

**Fig. 3:**
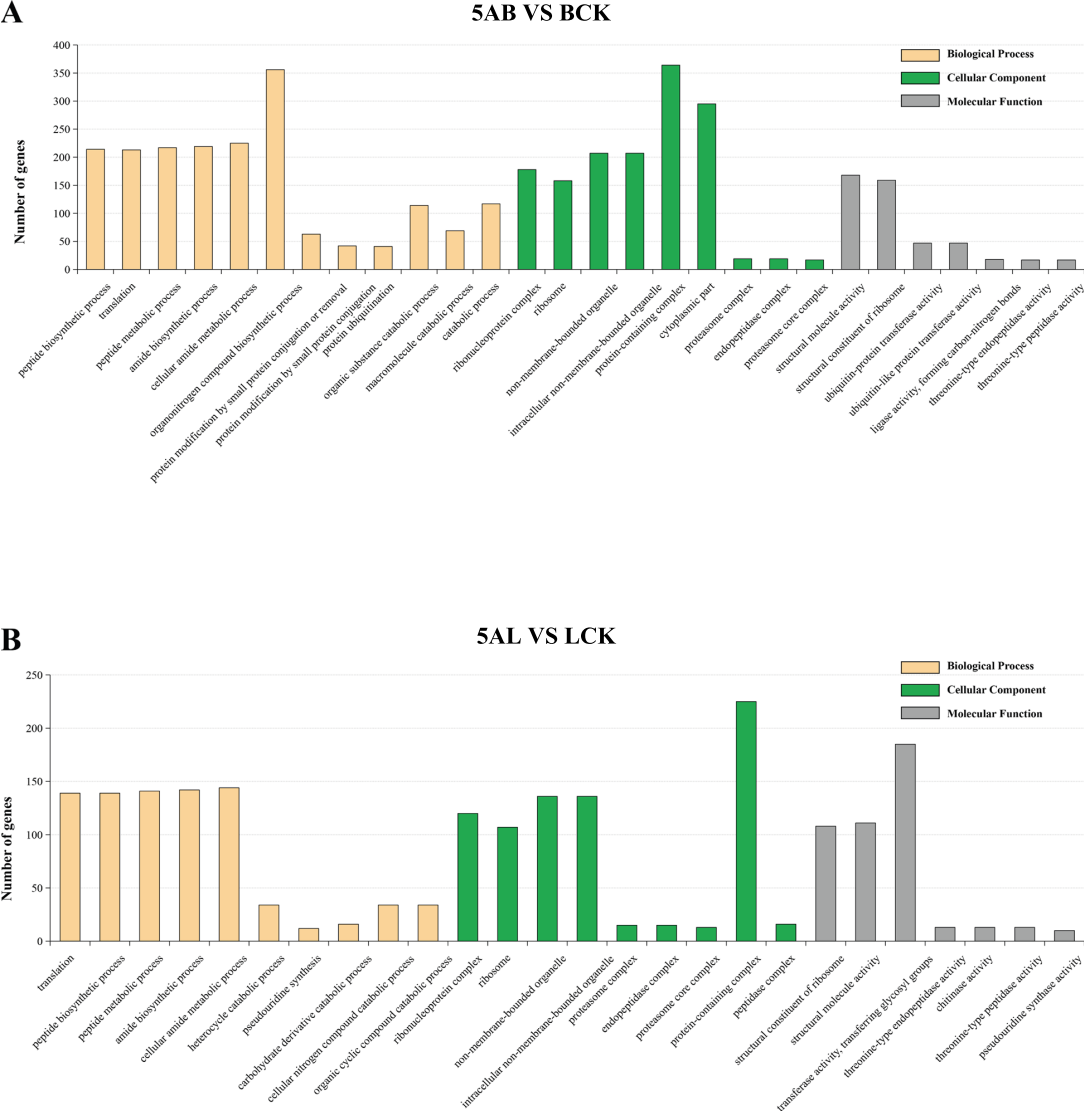
The classification of gene ontology (GO) and enrichment analysis of DEGs. GO terms are summarized in three functional groups: molecular function (grey), cellular component (green) and biological process (orange) in leaves and buds of ‘Kyoho’ grape after 5-azaC treatment. The vertical axis represents the number of enriched DEGs (padj < 0.05). **a** Buds, (**b**) leaves. 5AL; 5-azaC treated leaves, LCK; leaves control, 5AB; 5-azaC treated buds, BCK, buds control

The Gene Ontology (GO) knowledgebase is the world’s largest source of information and a comprehensive database on the functions of genes or proteins. GO consisted of three major domains, Biological Process (BP), Cellular Component (BP), and Molecular Function (BP) (Figs. S2A-C). GO annotation results for the buds treated with 5-azaC showed that DEGs were enriched for 12 annotations in biological processes, 9 annotations in cellular components, and 7 annotations in molecular functions (Table S2, Fig. 3a, Fig. S2C). The terms of “structural molecule activity” (GO: 0005198), “structural constituent of ribosome” (GO: 0003735), “ubiquitin-protein transferase activity” (GO: 0004842) and “ligase activity, forming carbon-nitrogen bonds” (GO: 0016879) were significantly enriched in the molecular function. The main enrich components of Biological processes were included “peptide biosynthetic process” (GO: 0043043), “translation” (GO: 0006412), “peptide metabolic process” (GO: 0006518), and “amide biosynthetic process” (GO: 0043604). In the cellular component, the enrich components were “ribonucleoprotein complex” (GO: 1990904), “ribosome” (GO: 0005840), and “non-membrane-bounded organelle” (GO: 0043228). The results of the 5-azaC treatment regarding the GO annotation were some extent similar to the previous results in leaves (Table S2, Fig. 3b, Fig. S2B). “Structural molecule activity” (GO: 0005198) and “structural constituent of ribosome” (GO: 0003735) were also the most enriched pathways in molecular functions, and also included “threonine-type endopeptidase activity” (GO: 0004298) and “transferase activity, transferring glycosyl groups” (GO: 0016757). The enriched terms in Biological processes and Cellular component were “peptide biosynthetic process” (GO: 0043043), “translation” (GO: 0006412), “peptide metabolic process” (GO: 0006518), “ribonucleoprotein complex” (GO: 1990904), and “Ribosome” (GO: 0005840) in leaves. These results were consistent with the obtained results of the bud after 5-azaC treatment. In addition, most of the DEGs belonged to the Biological processes were up-regulated, which suggest the involvement of 5-azaC in gene expression.

**Fig. 4:**
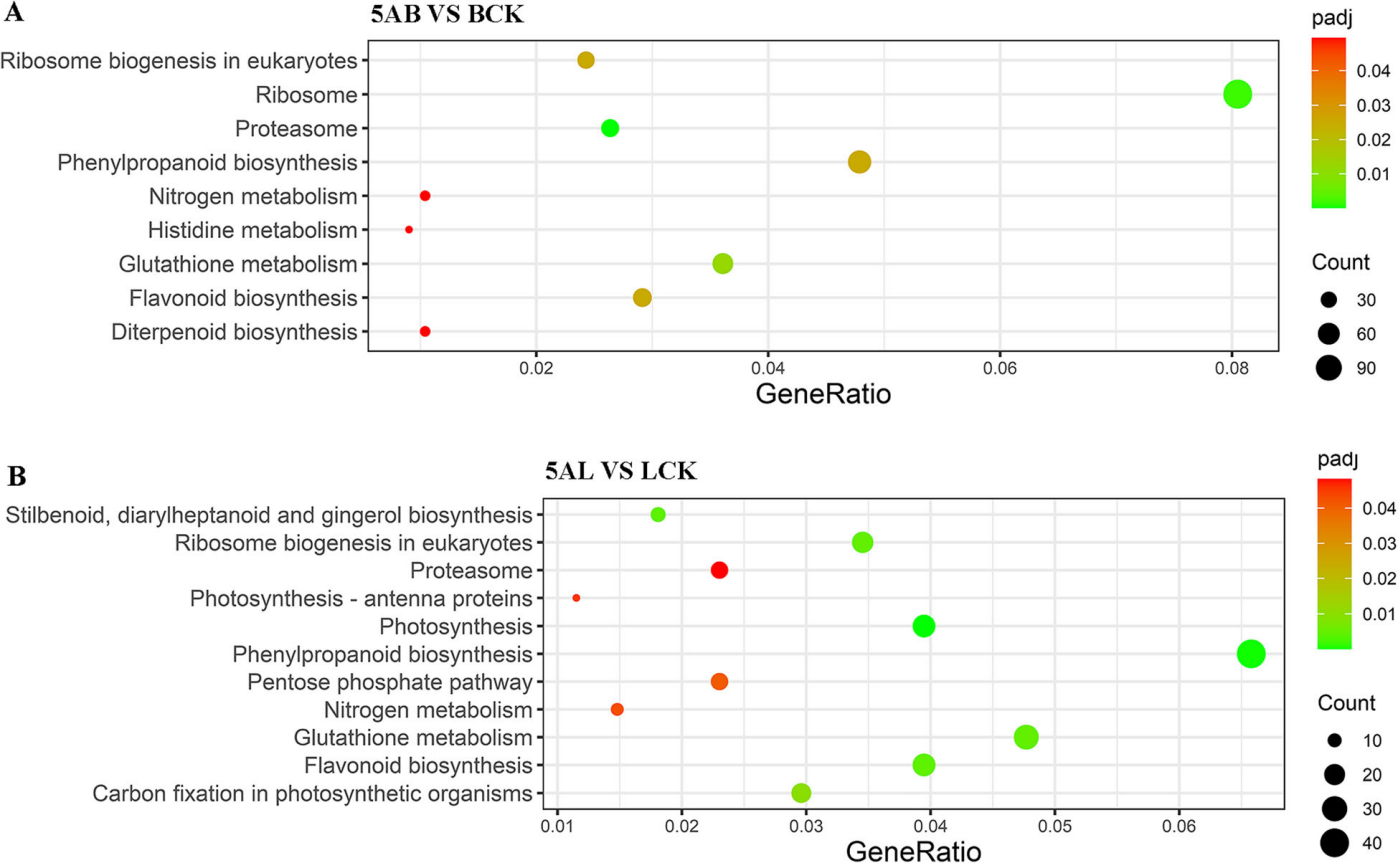
The KEGG pathway enrichment analysis of DEGs in buds (**a**) and leaves (**b**) of ‘Kyoho’ grape after 5-azaC treatment (padj < 0.05). Coloring indicates -log_10_(padj) with higher in red and lower in green. The lower padj indicates the most significantly enriched pathways. Point size indicates the number of DEGs. 5AL; 5-azaC treated leaves, LCK; leaves control, 5AB; 5-azaC treated buds, BCK; buds control

The Kyoto Encyclopedia of Genes and Genomes (KEGG) annotation was used to analyze the biological roles of DEGs that selected from the transcriptome data (Table S3). According to significance, we drew bubble diagrams for the significantly enriched KEGG pathways. In grape buds treated with 5-azaC, the biological pathways with the highest enrichment were “Proteasome” (vvi03050), “Ribosome” (vvi03010), “Glutathione metabolism” (vvi00480), “Ribosome biogenesis in eukaryotes” (vvi03008), “Flavonoid biosynthesis” (vvi00941), and “Phenylpropanoid biosynthesis” (vvi00940) (Fig. 4a). In leaves, we found “Photosynthesis” (vvi00195), “Phenylpropanoid biosynthesis” (vvi00940), “Flavonoid biosynthesis” (vvi00941) “Stilbenoid, diarylheptanoid and gingerol biosynthesis” (vvi00945), and “Ribosome biogenesis in eukaryotes” (vvi03008) as the most enriched and important biological pathways after 5-azaC exposure (Fig. 4b).

### Methylation positively regulated photosynthesis pathway

**Fig. 5:**
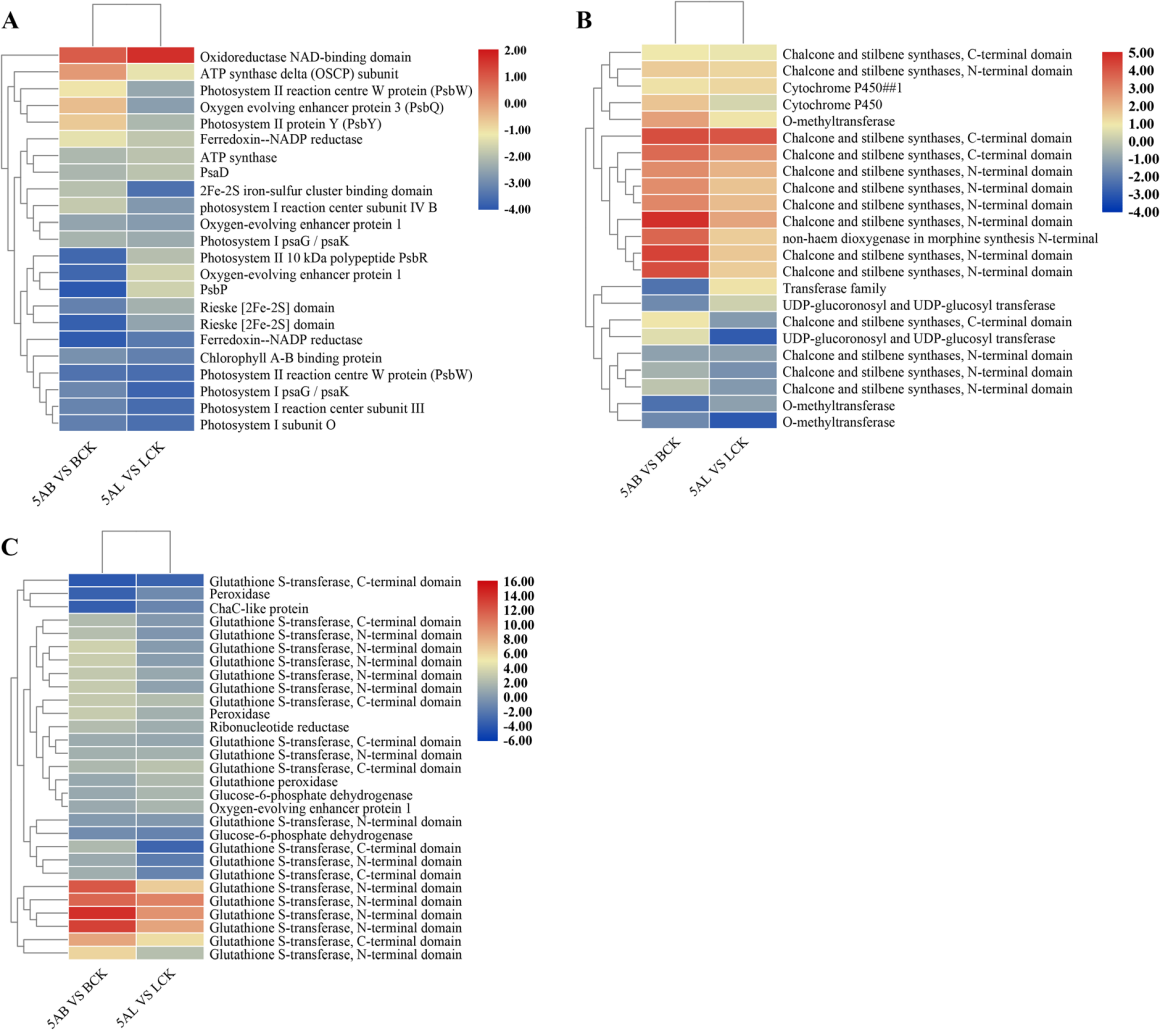
Heat maps of gene expression patterns involved in biological pathways obtained from KEGG. Heat maps were drawn according to log_10_ (Treatment FPKM/Control FPKM) in leaves and buds of ‘Kyoho’ grape. The rows and columns in the heat maps represent samples and genes, respectively. Red and blue represent the highest and lowest level of expression. **a** Photosynthesis, (**b**) Flavonoid biosynthesis, (**c**) Glutathione metabolism. 5AL; 5-azaC treated leaves, LCK; leaves control, 5AB; 5-azaC treated buds, BCK; buds control

Photosynthesis is one of the important biological processes in leaves that convert light energy into chemical energy [22]. According to the RNA-Seq results of leaves, we found 24 DEGs with significant enrichment in “Photosynthesis” (vvi00195) pathway, including photosystem I psaG/psaK, photosystem II reaction center W protein (PsbW), chlorophyll A-B binding protein, and oxygen evolving enhancer protein 3 (PsbQ) (Table S4, Fig. 5a, Fig. S3). Among photosynthetic responsive genes, 23 DEGs showed a downward trend after 5-azaC treatment, and only oxidoreductase NAD-binding domain showed an upward adjustment. This indicated that 5-azaC led to the suppression of photosynthesis genes and reduced the photosynthetic efficiency of the leaves. In addition, 2Fe-2S iron-sulfur cluster binding domain is a multi-subunit protein of iron binding and storage proteins, that down-regulation of this gene may inhibit leaf metabolic activities. This gene plays an important role in photosynthesis, growth, and development in the plant.

### Demethylation induced secondary metabolism

The KEGG annotation results depicted a large number of biosynthesis and metabolic processes including “Glutathione metabolism”, “Flavonoid biosynthesis”, and “Phenylpropanoid biosynthesis” (Table S4), which were significantly enriched in buds treated with 5-azaC. Flavonoids act as a secondary metabolic pathway in plants and accumulate in different organs including, leaves, roots, flowers, fruits, and seeds. Flavonoid biosynthesis pathway plays an important role in resistance against abiotic stress and diseases. Among key genes of flavonoids pathway, chalcone synthase, stilbene synthases, and UDP-glucosyl transferase were the most up-regulated, while Cytochrome P450 was down-regulated in response to 5-azaC (Table S4, Fig. 5b). In glutathione metabolism pathway, large number of glutathione S-transferases (*GST*) were identified, which regulate anthocyanin transport [23]. Out of 37 identified *GSTs*, 28 and 9 *GSTs* were significantly up- and down-regulated, respectively (Table S4, Fig. 5c). Phenylpropanoid biosynthesis was also enriched with a large number of DEGs, including 29 and 40 up- and down-regulated DEGs (Table S4). Phenylpropanoid compounds scavenge free radical scavenger and inhibit lipid peroxidation in grapes that lead to maintaining the redox balance in cells [24].

### Down-regulation of transcription factors occurred more under demethylation

**Fig. 6:**
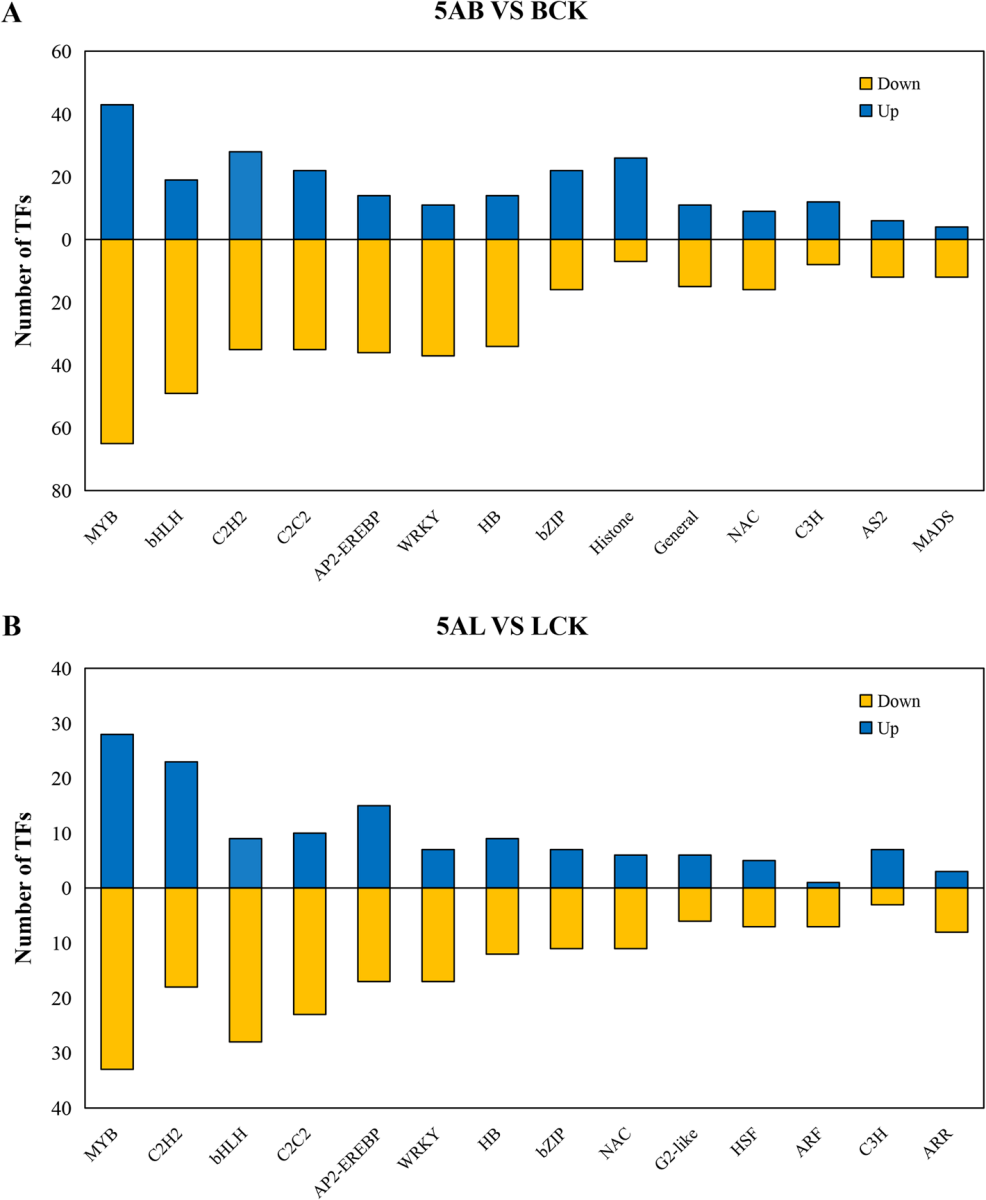
Determination of the top 14 transcription factor families in leaves and buds of ‘Kyoho’ grape after 5-azaC exposure. Blue represents the up-regulated TFs, yellow represents the down-regulated TFs, and the vertical axis shows the number of TFs. **a** Buds. **b** Leaves. 5AL; 5-azaC treated leaves, LCK; leaves control, 5AB; 5-azaC treated buds, BCK, buds control

Transcription factors (TFs) regulate the expression of gene networks through binding to cis-regulatory elements located in promoter regions of responsive genes that could be involved in plant metabolism, growth, and development. Transcriptome sequencing results showed that 906 and 494 TFs found in buds and leaves, respectively, which could be classified into 49 different transcription factor families. According to the transcriptome data from bud samples, the highest number of TFs belonged to different families including MYB (108, 11.92%), bHLH (68, 7.51%), C2H2 (63, 6.95%), and C2C2 (57, 6.29%). In leaves, TFs families were similar to buds in response to 5-azaC but were different in frequency (MYB (61, 10.32%), C2H2 (41, 8.30%), bHLH (37, 7.49%), and C2C2 (33, 6.68%)) families. We presented the top 14 transcription factor families by bar graphs and compared the up- and down-regulated TFs in treated buds and leaves with 5-azaC (Figs. 6a, b). We found that the MYB and bHLH families possess the highest members among TFs families in both leaves and buds, although, the number of down-regulated TFs was more than up-regulated TFs.

### Analysis of protein-protein interaction network

**Fig. 7:**
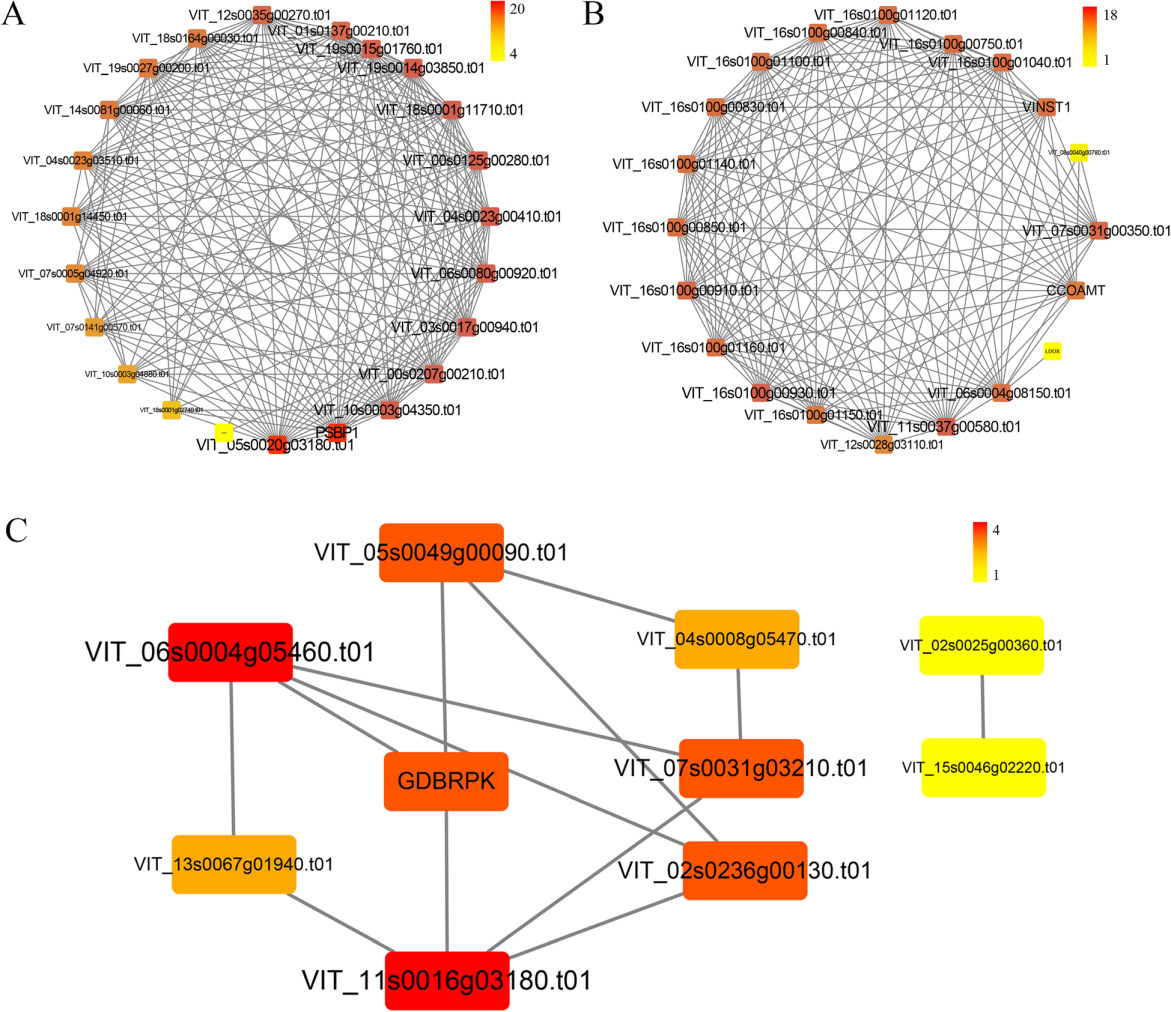
The protein-protein interaction networks were significantly enriched in the biological pathways including (**a**) photosynthesis, (**b**) flavonoid biosynthesis, and (**c**) MAPK signaling pathway. The node represents a certain gene, and the number of interacting proteins is displayed in the shades of color and the font size in leaves and buds of ‘Kyoho’ grape after 5-azaC exposure5AL; 5-azaC treated leaves, LCK; leaves control, 5AB; 5-azaC treated buds, BCK, buds control

We used STRING database to search the protein networks regarding functional enrichment analysis according to reported and predicted proteins. The obtained results were imported into Cytoscape for visual analysis [25]. To further study the regulatory networks and significantly enrich key pathways, we constructed protein-protein interaction for predicted DEGs. We found three networks regarding “Photosynthesis”, “Flavonoid biosynthesis”, and “MAPK signaling pathway”, which each node represents the number of genes (Figs. 7a-c). The color spectrum is used to indicate the number of nodal gene interaction proteins. In “Photosynthesis”, photosystem I reaction center subunit II is related to chloroplast photosynthesis rate, and it interacts with 20 proteins. *Leucoanthocyanidin dioxygenase* (*LDOX*) is a key enzyme at the end of the anthocyanin biosynthesis pathway, which catalyzes the conversion of colorless anthocyanins to colored anthocyanins [26], which only interacts with trans-cinnamate 4-monooxygenase. There are two separate paths in the ‘MAPK signaling pathway-plant’, that among them, the network of PP2C interaction is the most complicated. The PP2C mainly participated in multiple signal transduction pathways in plants, including abscisic acid, salicylic acid, and jasmonic acid, although it has an important regulatory role in other pathways [27, 28]. In summary, photosystem I reaction center subunit II, caffeoyl-CoA O-methyltransferase, and protein phosphatase 2C are the key genes in the regulatory networks.

### Validation of DEGs using qPCR

**Fig. 8:**
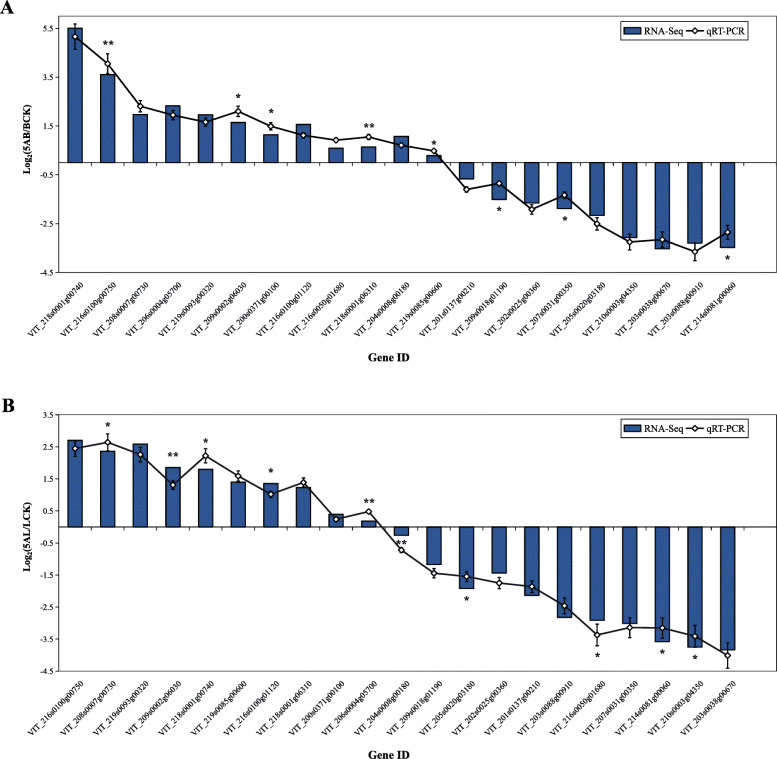
The validation of RNA-seq data using qRT-PCR in the selected DEGs. Relative expression ratio of each DEG is presented in a log_2_ (Fold Change). The values are mean ± SE and error bars represent standard deviations from three independent technical replicates. The asterisk indicates the significance level based on the independent samples t test (* *P* < 0.05, ** *P* < 0.01). **a** Buds, (**b**) leaves

To validate the accuracy and reproducibility of RNA-Seq data, we analyzed the expression levels of related genes in buds and leaves by qPCR after 5-azaC treatment (Figs. 8a, b). We selected 21 DEGs for qPCR that participated in different biological pathways including “Photosynthesis”, “Phenylpropanoid biosynthesis”, “Flavonoid biosynthesis”, “Glutathione metabolism”, “Ribosome”, “Carbon fixation in photosynthetic organisms”, and “MAPK signaling pathway”. According to the results of qPCR, we found that it has a good linear relationship with the transcriptome data, and the correlation coefficient (R^2^) in buds and leaves were 0.9727 and 0.9798, respectively. Therefore, these results confirmed the accuracy and reliability of the RNA-Seq dataset.

## Discussion

DNA methylation is an epigenetic modification required for the silencing, gene regulation, and genomic imprinting of transposable elements (TE) [29]. In the present study, we used RNA-Seq as a high throughput sequencing technology to explore the specific effects of reduced methylation levels on grape leaves and buds after 5-azaC treatment. Through KEGG and GO enrichment analysis, key genes and biological pathways were screened to comprehensively analyze the specific effects of 5-azaC on gene expression and regulatory networks. DNA methylation is a form of genome epigenetic inheritance, which affects the expression of genes. 5-azaC is a cytosine nucleoside analogue that inhibits the DNA methylation process by binding to methyltransferases and reduce the genome methylation level. In our previous study, 5-azaC was sprayed on ‘Kyoho’ fruit during color transformation, we found that the decrease in methylation level suppressed the anthocyanin pathway, cell wall metabolism, and aroma-related gene expression by bisulfite sequencing PCR (BSP). The results indicated that demethylation may delay the fruit ripening process [19]. As a methylation inhibitor, 5-azaC has been used to reduce the genome methylation level in various plants. This is evident from previous studies, that changes in methylation level affect the growth and development in plants [18, 30]. The 5-azaC treatment also induced flowering earlier than control in Arabidopsis and demonstrated that DNA methylation plays a critical role in preventing early flowering in Arabidopsis [31]. Additionally, the changes in the methylation levels have positive or negative effects in response to biotic and abiotic stresses. Researchers have focused on the regulation of fruit maturation and ripening through methylation levels. In tomato, hypermethylation inhibited fruit ripening through repression of TFs involved in ripening and rate-limiting enzymes of carotenoid synthesis [19]. The large number of genes related to inhibiting or regulating maturation in the fruits of *SlDML2* tomato mutants, which is a DNA demethylase, regulated gene expression [19]. During the ripening process of Orange fruit, the expression of DNA demethylase genes decreased and the methylation levels of the genome increased [20]. Furthermore, 5-azaC treatment of ‘Kyoho’ grapes at different developmental stages of fruits led to the differences in transcript expression levels [32]. Although 5-azaC treatment has not influenced the expressions of most genes but DEGs could be involved mainly in fruit softening, photosynthesis, protein phosphorylation, and heat stress [32]. Meanwhile, there are many types of researche about methylation affecting plant growth and development but the regulating mechanism is not yet clear in other tissues, and the effect of methylation in the regulatory networks needs to be investigated by more extensive experiments.

### Changes in photosynthesis-related genes

Leaves are the main source of photosynthesis and metabolism to produce metabolites for plant survival [33]. The transcriptome results showed that 23 out of the 24 genes belonged to photosynthesis were suppressed, and the only single transcript was up-regulated. These genes participated in photosystem PSI and PSII such as Photosystem I psaG /psaK and Photosystem II reaction center W protein (PsbW), although PSII will respond earlier than PSI in response to stress by damage of D1 protein [34]. Meanwhile, D1 protein can quickly repair and restore its activity under the light. It has been shown that salt stress causes PSII damage and reduce the maximum photochemical efficiency of PSII [35]. The PSI will also be damaged under various stress conditions, and recovery will be extremely slow or even irreversible after the stress [36]. Under high-temperature conditions, PSII limits the transfer process of photosynthetic electrons to PSI, reduces the production of active oxygen, and hinders the photosynthetic efficiency. Through the analysis of photosynthesis metabolic pathways (Figs. S4A,B), the enzyme activities involved in photosynthesis were also inhibited in response to 5-azaC. CO_2_ assimilation is another important aspect of the photosynthesis process [37]. Therefore, demethylation also suppressed the activity of all genes regarding Calvin cycle, resulting in a reduction of photosynthetic products and organic matter [38]. Although, our results pointed to 5-azaC inhibitory effect on leaf photosynthesis, the mode of action of carbon assimilation remains to be explored.

### Response of hormone-related genes to methylation

Plant hormones including auxin, ethylene, abscisic acid (ABA), gibberellic acid (GA), and jasmonate (JA) have regulatory functions for various biological processes, such as metabolism, growth, and development in grapes [39, 40]. Dormancy of buds is a vital developmental process and a mechanism for the defense against adverse environments [15]. The initiation, termination, and bud dormancy are generally controlled by endogenous hormones [41]. As a typical non-climacteric fruit, ethylene has no obvious changes during the developmental stage. Studies have proved that ethylene induces the dormancy of grape buds [3, 42]. Spraying ethylene on buds causes dormancy earlier or ends dormancy later. Ethylene insensitive 3 (EIN3) is a key TF in the ethylene signaling pathway and has a regulatory effect on downstream ethylene response factor (ERF) [43]. According to our transcriptome dataset regarding buds, four EIN3 were screened that three of them significantly down-regulated. Therefore, it showed that the expression of EIN3 is inhibited by 5-azaC. Gibberellin (GA) can break the dormancy of grape buds and promotes germination. The increase in GA content is considered to be a key factor for bud germination [44]. The ten gibberellin regulated-transcripts were strongly inhibited, which may lead to the reduction of endogenous GA content in buds by demethylation. In addition, ABA is the main inhibitory factor for bud germination [45], which low ABA content makes buds more likely to germinate [46]. ABA is also associated with leaf shedding that synthesized and transported to other parts of the leaves. Proper cutting can reduce ABA synthesis, decrease the ABA accumulation in bud tissue, and reduce the dormancy level to facilitate germination [47]. In our study, auxin responsive transcripts frequently appeared, although the mechanism of auxin for bud dormancy is not yet clear. Some studies have pointed out that GA/ABA ratio indirectly regulates the germination, however, the application of exogenous auxin positively affects seed germination [48–50] (Table S5). Hormones cooperate with each other in plants to regulate various biological processes, and we still need in-depth experiments to further explore the mechanism of grape germination.

### Cell wall and sucrose metabolism after 5-azaC treatment

The cell wall functions to maintain cell morphology and ensure the normal exchange of material between cells. The rate of cell wall degradation depends on the activity of metabolic enzymes in the cell wall. We determined the key enzymes of cell wall metabolism including polygalacturonase (PG), pectin methylesterase (PME), and pectinesterase (PE), which showed a general decline in expression, indicating 5-azaC inhibited the degradation of the cell wall. Sucrose is produced by photosynthesis and provides the necessary energy for plant growth and development [51]. Previous studies demonstrated that sucrose and ABA coordinately regulated ripening, and sucrose could be used as a signaling molecule to induce the expression of ripening-related genes [7, 52]. In the early growth stages of *Arabidopsis thaliana*, sucrose acts as a long-distance signal to regulate root growth [53]. In sucrose metabolism pathway, the sucrose synthase and sucrose phosphate synthase might be coordinated with other functional genes to regulate the growth and development of buds and leaves (Figs. S5A,B).

### Down-regulation of regulatory factors by demethylation

TFs bind to specific DNA sequences of promoters to activate or suppress the transcription of genes and regulate expression levels [54]. A large number of TFs families were differentially expressed in transcriptome levels, including members belonged to MYB, bHLH, C2H2, and AP2-EREBP. In general, MYB and bHLH are involved in flavonoid synthesis pathway of grape, which regulate the accumulation of anthocyanins and form color [55]. Under drought conditions, MYB2 and bHLH2 act as transcription activators in ABA signaling pathway [53, 56]. In addition, the transcriptional activity of MYB and bHLH under Fe-deficiency conditions affects the tolerance against stress [57]. In this study, after the leaves were treated with 5-azaC, most of transcripts belonged to MYB and bHLH families were down-regulated. Furthermore, WRKY family plays an important role in the plant defense signaling network and can induce the gene expression through W box elements in response to fungal elicitors [58]. In this study, most members of WRKY family were down-regulated in response to 5-azaC, therefore, demethylation regulates various biological activities and metabolic processes through down-regulation of TFs (Figs. S6A,B).

### Analysis of MAPK signaling pathway

The MAPK signaling pathway is involved in environmental stresses and monitors growth and development through signal transduction [59]. A study demonstrated MEKK1-MKK4/5-MPK3/6-WRKY22/29/33 cascade path participated in the disease resistance in *Arabidopsis thaliana*, and MPK/MPK6 could activate the expression of WRKY33 and promote the synthesis of photoprotections [60]. Furthermore, MAPK4 plays an important role in the signal’s transduction under adverse stimuli, which significantly increased under salt stress, low temperature, and sucrose starvation [61]. In addition, MAPK has bifunctional roles and act as a positive and negative regulator in response to ABA, auxin, and JA [62]. In our analysis, the genes of MAPK signaling pathways were enriched, which consisted of pathogenesis-related proteins (PRs). PRs are an important component of the plant defense system against biotic stress [63]. We observed that PR1 was significantly suppressed by 5-azaC, indicating that demethylation affects grape resistance. Heat shock proteins (HSPs) in plants function in acclimation to environmental temperature. For example, overexpression of HSPs made rice drought and heat stress-tolerant [64]. It is worth noting that almost all the HSPs in leaves and buds were significantly inhibited, therefore, demethylation interfered with the expression of HSPs and weakened the heat adaptability. The specific mechanism of action still needs to be revealed by further experiments.

## Conclusions

**Fig. 9:**
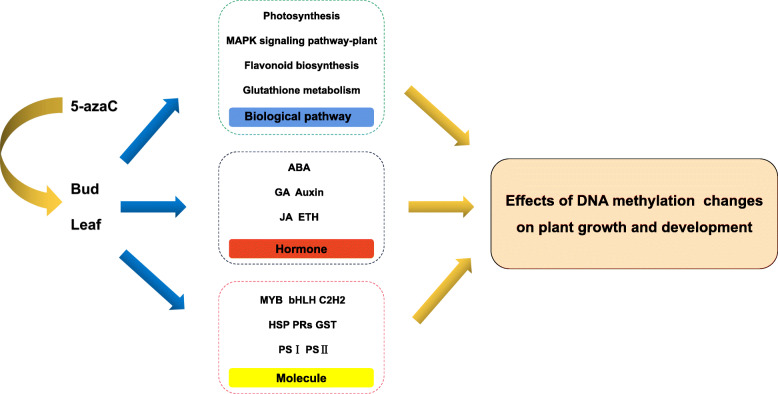
Schematic model of DNA methylation effects on the regulatory networks of leaves and buds of ‘Kyoho’ grape including biological pathways, hormone, and molecular pathways

Based on our current research on ‘Kyoho’ fruits, we performed RNA-Seq analysis to compare the transcriptome profiles in the buds and leaves and investigate the gene expression network in various metabolism pathways after 5-azaC treatment (Fig. 9). We identified many DEGs in bud (8050 genes) and leaf (3571 genes) and found that 5-azaC has different effects on various tissues. Functional enrichment analysis indicated that 5-azaC treatment on buds was mainly affected the pathways of ‘Proteasome’, ‘Ribosome’, and ‘Glutathione metabolism’, while the altered pathways in the leaves were ‘Photosynthesis’, ‘Phenylpropanoid biosynthesis’, and ‘Flavonoid biosynthesis’. Overall, functional analysis showed that 5-azaC could inhibit certain metabolic pathways, which can have a negative impact on biological processes. According to protein-protein interaction networks, we discovered that photosystem I reaction center subunit II, caffeoyl-CoA O-methyltransferase, and protein phosphatase 2C are the key genes in the regulatory networks. In summary, we demonstrated that demethylation regulated the molecular mechanisms and regulatory networks in grape, and possibly accumulate the specialized metabolites.

## Methods

### Plant material

All leaves and buds were collected from ‘Kyoho’ grapevine (*Vitis labrusca* L. ˟ *V. vinifera*) from the Jiangsu Academy of Agricultural Sciences (located at 31 ^o^N Lat. and 118 °E Long.) Nanjing, China. The ungerminated buds and newly grown leaves of ‘Kyoho’ grapevine were collected in the same growth stage, which were free of diseases and pests. We selected plant materials using phenotypic observation and quality determination. The samples were treated with 0.01% Triton X-100 and 20 mM 5-azaC (Sigma) solution, sprayed once every 2 days for a total of 3 times. Seven days after the last treatment, the samples were collected, frozen in liquid nitrogen, and stored at − 80 °C. Distilled water was used as a negative control. Each treatment was repeated three times.

### RNA extraction, cDNA library construction and Illumina sequencing

Total RNA from grape leaves and buds were extracted and purified using QIAGEN RNeasy Plant Mini Kit (QIAGEN, Germany) and manufacturer’s instructions. To digest DNA and remove interference, we added DNase (Takara, China). RNA concentration was measured by Thermo Scientific™ NanoDrop™ spectrophotometer (Thermo Scientific, Wilmington, DE, USA). Further mRNA purification and cDNA library construction were performed with the Ultra II RNA Library Prep Kit for Illumina (NEB, USA). All samples were sequenced on the Illumina Hiseq™ 2500 (Beijing Nuohe Zhiyuan Technology Co., Ltd. Beijing, China).

### Analysis of RNA-sequence data

To ensure data quality, we used HISAT (Version: v2.1.0) to filter and align the reads, and mapped the clean reads with the grape gene sequence reference datasets by Bowtie2 (Version: v2.2.5) [65]. RSEM (Version: v1.2.8) was used to calculate the fragments per kilobase of exon per million mapped reads (FPKM) to estimate transcript abundance within 95% confidence interval [66]. We calculated the *P* value according to the negative binomial distribution, and finally performed the multiple hypothesis test correction (BH) to obtain the FDR (false discovery rate). FDR ≤ 0.05 and |log_2_(Fold change) | > 1 were defined as statistically significant.

### Enrichment analysis

In order to obtain more detailed information about DEGs, we performed enrichment analysis using R packages (Version: 3.18.1) [67] and annotated DEGs using gene ontology (GO) (http://geneontology.org/) and Kyoto Encyclopedia of Genes and Genomes (KEGG) (https://www.genome.jp/kegg/) databases. We set the threshold at padj < 0.05 to determine the significant enrichment of KEGG pathways and GO terms.

### Visualization of metabolism pathways using Mapman

*Vitis vinifera* Gene Index sequences were download from an online website (https://www.plabipd.de/portal/mercator-sequence-annotation). We extracted the Gene ID and log_2_ (Fold Change) data lists in the transcriptome data, and used Mapman (version: MapMan 3.5.1R2) to visually analyze the ‘Metabolism overview’, ‘Photosynthesis’, ‘Biotic stress’, and ‘Flavonoid’ pathways.

### Construction and analysis of protein-protein interaction network

To further study the regulatory network of DEGs in key biological pathways, we used STRING (Version: 11.0) (https://string-db.org/) to search the interaction of proteins and find core regulatory proteins. The results of interaction proteins were imported into Cytoscape (Version: 3.6.1) to visually analyze the interaction network of genes.

### Validation of RNA–Seq data by qPCR

To validate the expression pattern of screened DEGs, qPCR was performed. According to Porebski et al. [68], the total RNA of each sample was extracted using Hexadecyltrimethylammonium Bromide (CTAB) method. We used PrimeScript™ IV 1st strand cDNA Synthesis Mix (Takara, Dalian, China) to synthesize cDNA from RNA, and diluted 5 times in ddH_2_O as a template. Primers for 21 genes were designed with the online software Primer3 Plus (http://primer3.ut.ee/) (Table S6). The qPCR was performed in a two-step method on Thermal Cycler Dice™ Real Time System III (Code No. TP950) (Takara, Dalian, China). Each reaction solution consisted of 10.0 μl of SYBR Premix Ex Taq™ (Takara, Japan), 0.4 μl of each primer (10 μM), 2 μl of cDNA, and 7.2 μl of RNase-free water in a total volume of 20 μl. The qPCR cycle parameters were set at 95 °C for 2 min, 95 °C for 10 s, and annealing temperature at 60 °C for 40 s, which comprised a total of 40 cycles. We selected grape gene *Actin1* (GenBank Accession number AY680701) as a reference for gene expression analysis. We calculate the relative expression of mRNA using 2^-ΔΔCT^ equation [69].

## Supplementary Information

**Additional file 1: Fig. S1.** Cluster analysis of DEGs using heat map in leaves and buds of ‘Kyoho’ grape after 5-azaC exposure. Genes or samples with similar expression patterns are clustered together. Red and blue represent the highest and lowest level of expression. 5AL; 5-azaC treated leaves, LCK; leaves control, 5AB; 5-azaC treated buds, BCK, buds control. **Fig. S2.** GO enrichment analysis of the specific and non-specific DEGs in leaves and buds of ‘Kyoho’ grape after 5-azaC exposure. (A) Non-specific DEGs, (B) Specific genes in leaves, and (C) buds. GO is divided into three major functional categories: molecular function (yellow), cellular component (blue), and biological process (red). The vertical axis represents the number of enriched DEGs (padj < 0.05). **Fig. S3.** DEGs involved in the photosynthetic pathway under 5-azaC treatment. The vertical axis is the name of DEGs, and the horizontal axis is the difference multiple according to fold change. **Fig. S4.** Visual analysis of DEGs in the photosynthesis pathway including light reactions, calvin cycle, and photorespiration in leaves (A) and buds (B) of ‘Kyoho’ grape after 5-azaC exposure. Each square represents a gene, green represents down regulation, and red represents up regulation. **Fig. S5.** Visual analysis of ‘Metabolism overview’ by Mapman in leaves (A) and buds (B) of ‘Kyoho’ grape after 5-azaC exposure. Changes in multiple metabolic pathways was observed, such as starch metabolism, sucrose metabolism, cell wall metabolism and lipid metabolism. Each square represents a gene, and red to green gradient represents up to down regulation. **Fig. S6.** Changes in DEGs expression during responses of plant to biotic stress in leaves (A) and buds (B) of ‘Kyoho’ grape after 5-azaC exposure. The changes of DEGs expression are appeared by the color of the grid, which green represents down regulation, while red represents up regulation.

**Additional file 2: Table S1.** All the identified DEGs in bud and leaf. **Table S2.** GO enrichment analysis of DEGs. **Table S3.** KEGG enrichment analysis of DEGs of grapevine buds and leaves. **Table S4.** List of DEGs in different metabolism pathways. **Table S5.** List of hormone-related DEGs. **Table S6.** List of primers for qRT-PCR analysis.

## Data Availability

All data generated or analyzed during this study are included in this published article [Supplementary Tables S1-S6]. Any reasonable requests are available from the corresponding authors (745896321@qq.com and jiahaifeng@njau.edu.cn).

## Abbreviations

*5AB*: 5-azacytidine treated buds
*5AL*: 5-azacytidine treated leaves
*5-azaC*: 5-azacytidine
*BCK*: Buds control
*BSP*: Bisulfite Sequencing PCR
*CTAB*: Hexadecyltrimethylammonium Bromide
*DEGs*: Differentially expressed genes
*EIN3*: Ethylene insensitive 3
*FDR*: False discovery rate
*FPKM*: Fragments Per Kilobase of transcript per Million mapped reads
*GST*: Glutathione S-transferases
*HISAT*: Hierarchical Indexing for Spliced Alignment of Transcripts
*HSP*: Heat shock protein
*IBA*: Indole-3-Butytric acid
*LCK*: Leaves control
*LDOX*: Leucoanthocyanidin dioxygenase
*PCA*: Principal Component Analysis
*PE*: Pectinesterase
*PG*: Polygalacturonase
*PME*: Pectin methylesterase
*PP2C*: Protein phosphatase 2C
*PR*: Pathogenesis-related protein
*qPCR*: Quantitative real-time PCR
*TF*: Transcription factor
