# Supplementary material for: Demethylation alters transcriptome profiling of buds and leaves in ‘Kyoho’ grape
Source: BMC Plant Biol. 2020 Dec 4;20:544. doi: 10.1186/s12870-020-02754-0 (PMC7716455; doi:10.1186/s12870-020-02754-0)
Supplement: Supplementary file 1 — Additional file 1: Fig. S1. Cluster analysis of DEGs using heat map in leaves and buds of ‘Kyoho’ grape after 5-azaC exposure. Genes or samples with similar expression patterns are clustered together. Red and blue represent the highest and lowest level of expression. 5AL; 5-azaC treated leaves, LCK; leaves control, 5AB; 5-azaC treated buds, BCK, buds control. Fig. S2. GO enrichment analysis of the specific and non-specific DEGs in leaves and buds of ‘Kyoho’ grape after 5-azaC exposure. (A) Non-specific DEGs, (B) Specific genes in leaves, and (C) buds. GO is divided into three major functional categories: molecular function (yellow), cellular component (blue), and biological process (red). The vertical axis represents the number of enriched DEGs (padj < 0.05). Fig. S3. DEGs involved in the photosynthetic pathway under 5-azaC treatment. The vertical axis is the name of DEGs, and the horizontal axis is the difference multiple according to fold change. Fig. S4. Visual analysis of DEGs in the photosynthesis pathway including light reactions, calvin cycle, and photorespiration in leaves (A) and buds (B) of ‘Kyoho’ grape after 5-azaC exposure. Each square represents a gene, green represents down regulation, and red represents up regulation. Fig. S5. Visual analysis of ‘Metabolism overview’ by Mapman in leaves (A) and buds (B) of ‘Kyoho’ grape after 5-azaC exposure. Changes in multiple metabolic pathways was observed, such as starch metabolism, sucrose metabolism, cell wall metabolism and lipid metabolism. Each square represents a gene, and red to green gradient represents up to down regulation. Fig. S6. Changes in DEGs expression during responses of plant to biotic stress in leaves (A) and buds (B) of ‘Kyoho’ grape after 5-azaC exposure. The changes of DEGs expression are appeared by the color of the grid, which green represents down regulation, while red represents up regulation. [file 12870_2020_2754_MOESM1_ESM.docx]

**Supporting information**


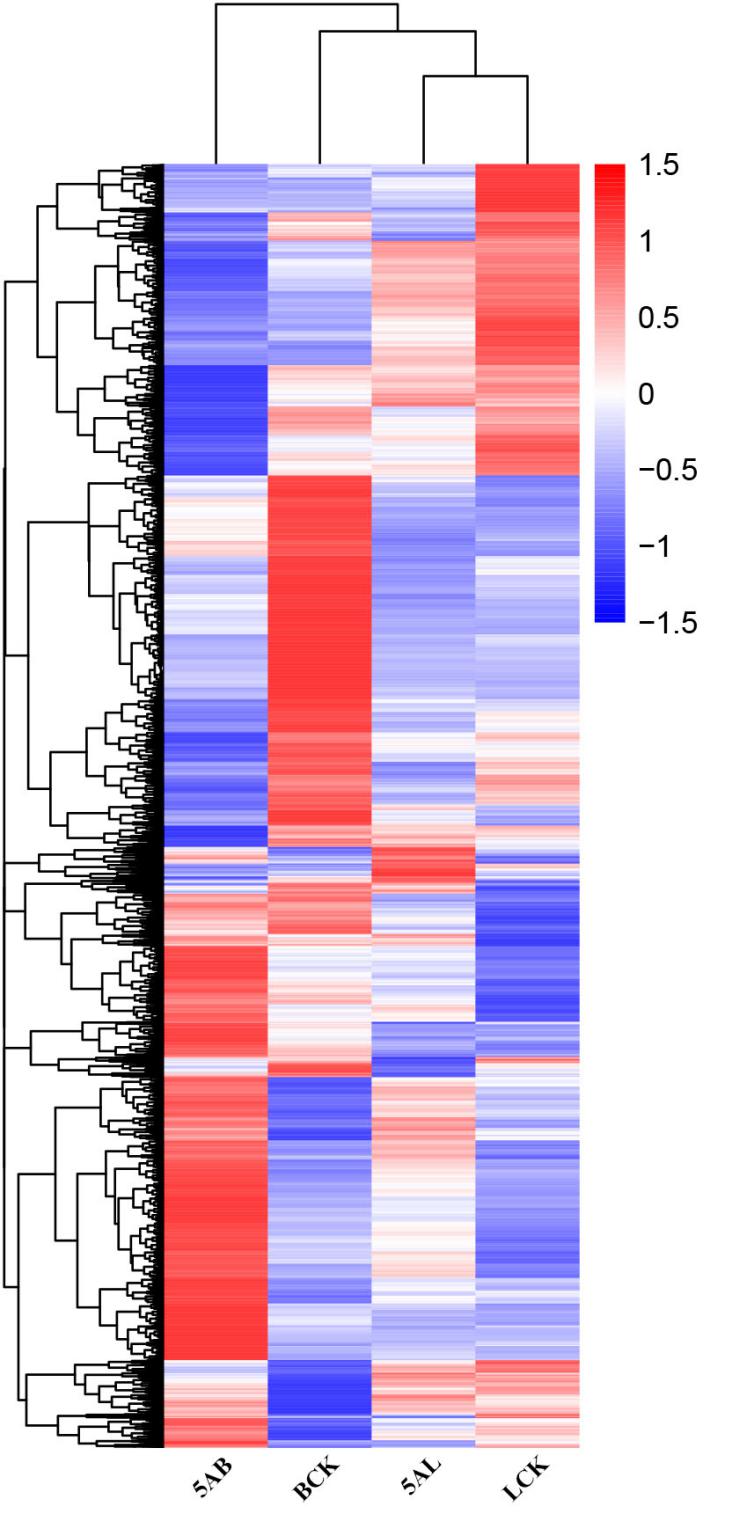


**Fig. S1**

Cluster analysis of DEGs using heat map in leaves and buds of ‘Kyoho’ grape after 5-azaC exposure. Genes or samples with similar expression patterns are clustered together. Red and blue represent the highest and lowest level of expression. 5AL; 5-azaC treated leaves, LCK; leaves control, 5AB; 5-azaC treated buds, BCK, buds control.


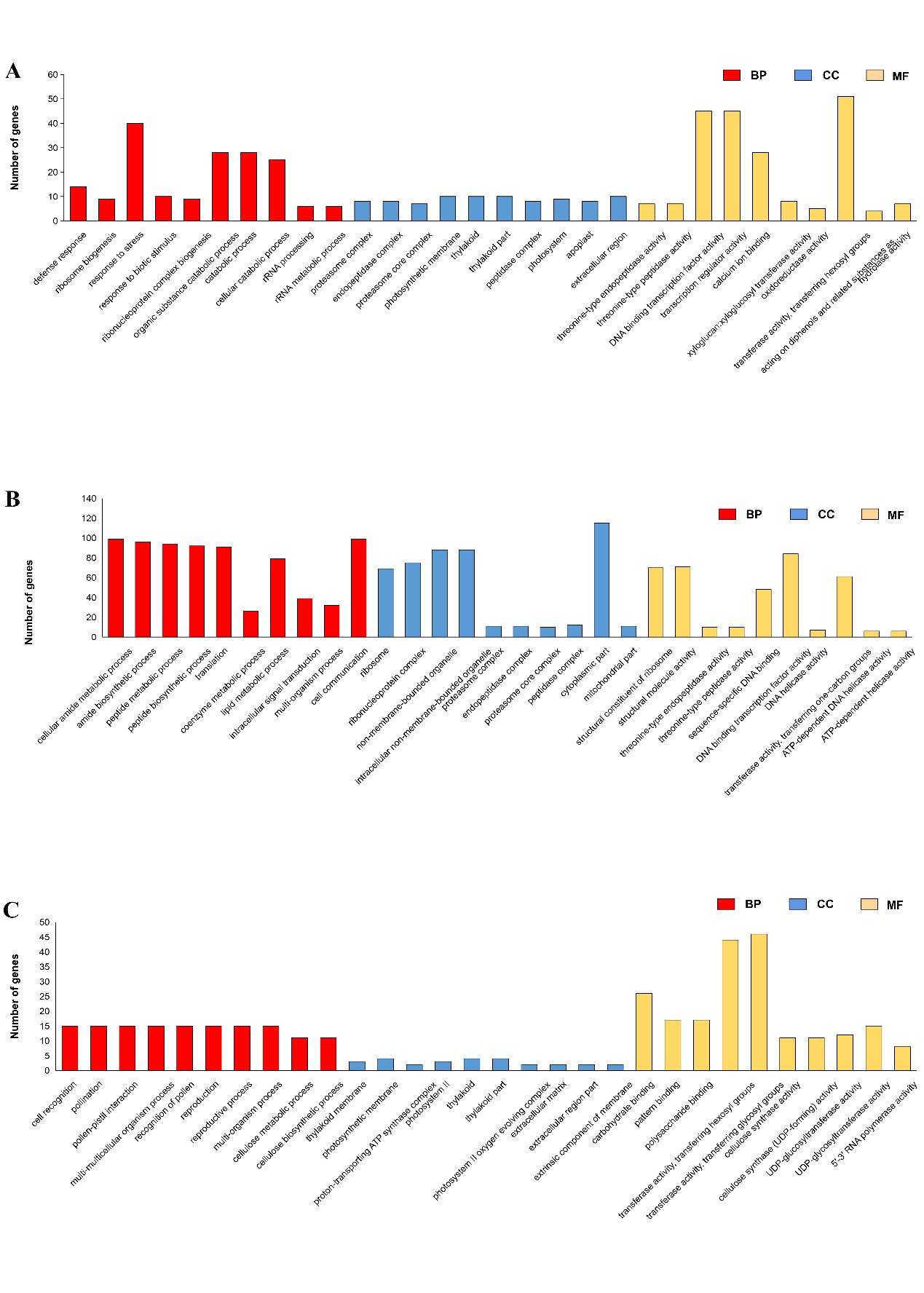


**Fig. S2**

GO enrichment analysis of the specific and non-specific DEGs in leaves and buds of ‘Kyoho’ grape after 5-azaC exposure. (A) Non-specific DEGs, (B) Specific genes in leaves, and (C) buds. GO is divided into three major functional categories: molecular function (yellow), cellular component (blue), and biological process (red). The vertical axis represents the number of enriched DEGs (padj < 0.05).


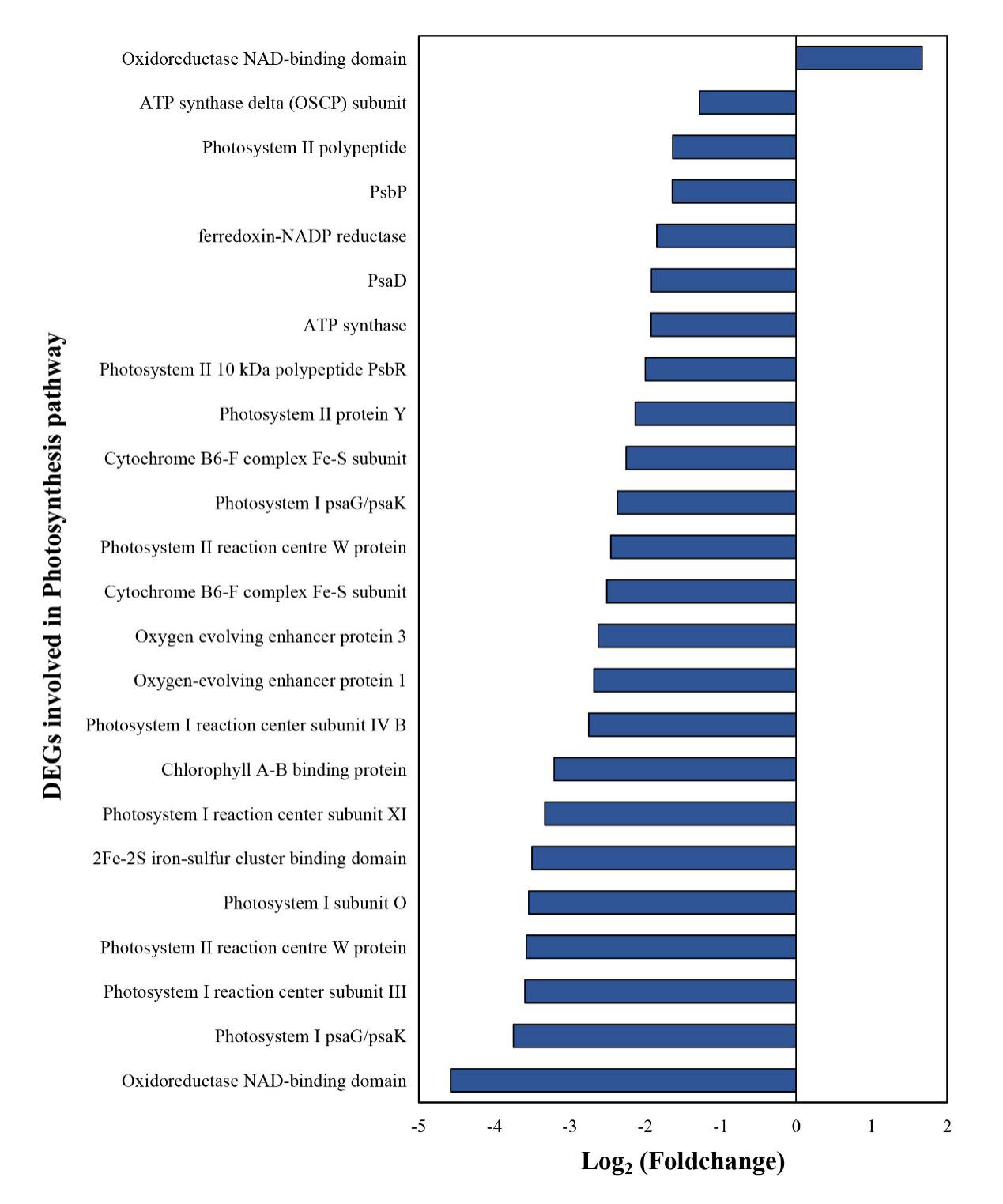


**Fig. S3**

DEGs involved in the photosynthetic pathway under 5-azaC treatment. The vertical axis is the name of DEGs, and the horizontal axis is the difference multiple according to fold change.


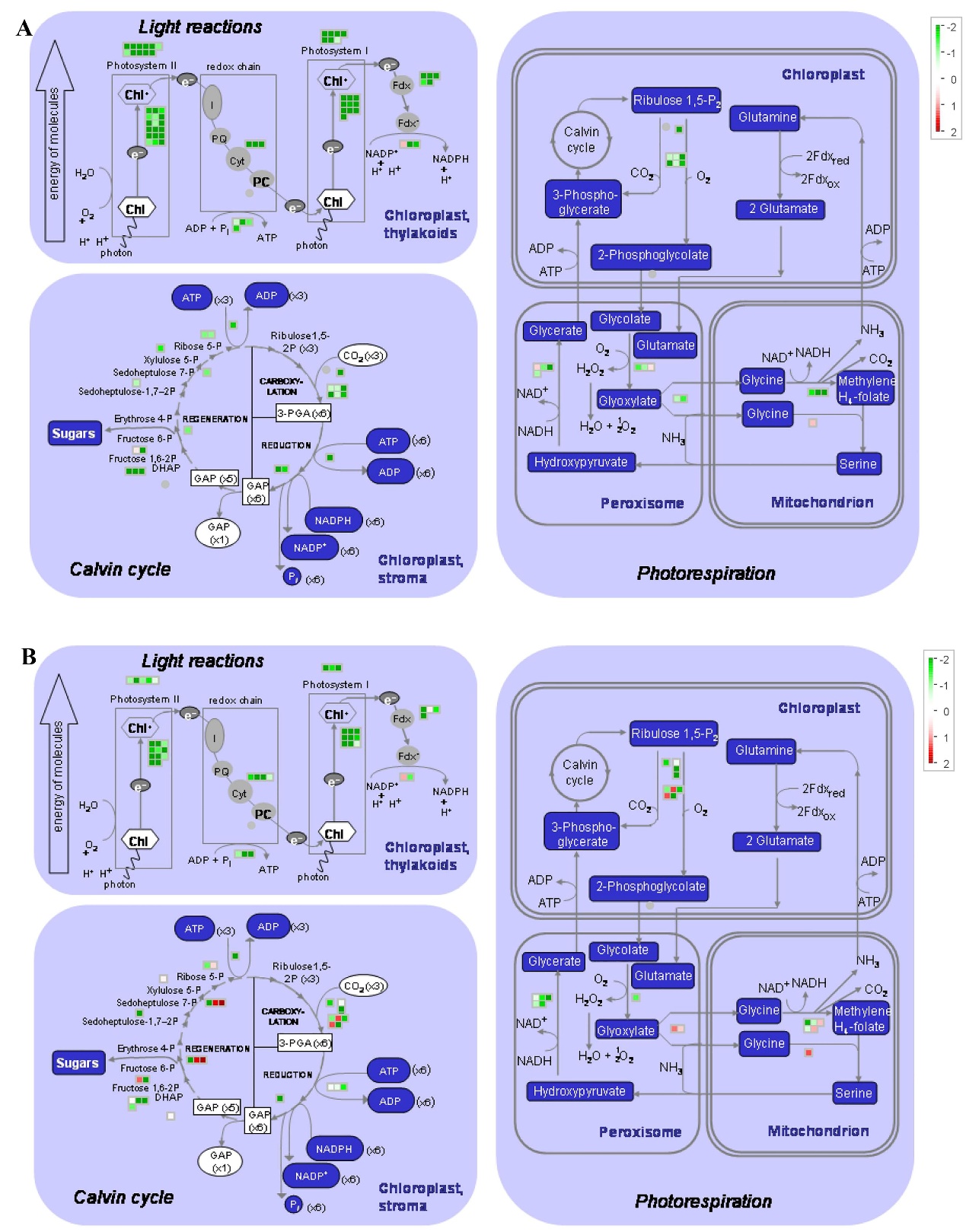


**Fig. S4**

Visual analysis of DEGs in the photosynthesis pathway including light reactions, calvin cycle, and photorespiration in leaves (A) and buds (B) of ‘Kyoho’ grape after 5-azaC exposure. Each square represents a gene, green represents down regulation, and red represents up regulation.


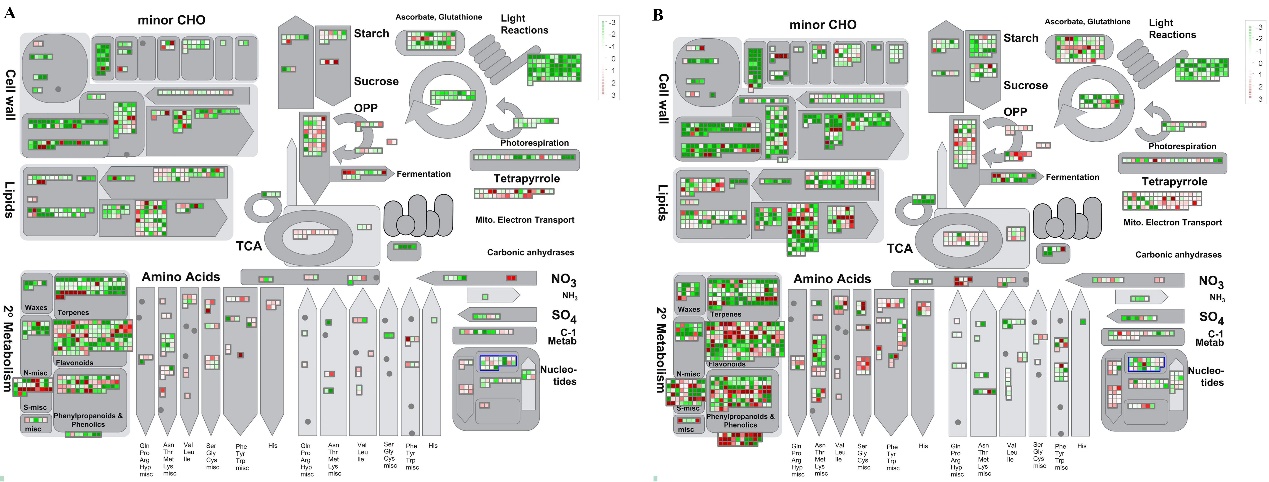


**Fig. S5**

Visual analysis of ‘Metabolism overview’ by Mapman in leaves (A) and buds (B) of ‘Kyoho’ grape after 5-azaC exposure. Changes in multiple metabolic pathways was observed, such as starch metabolism, sucrose metabolism, cell wall metabolism and lipid metabolism. Each square represents a gene, and red to green gradient represents up to down regulation.


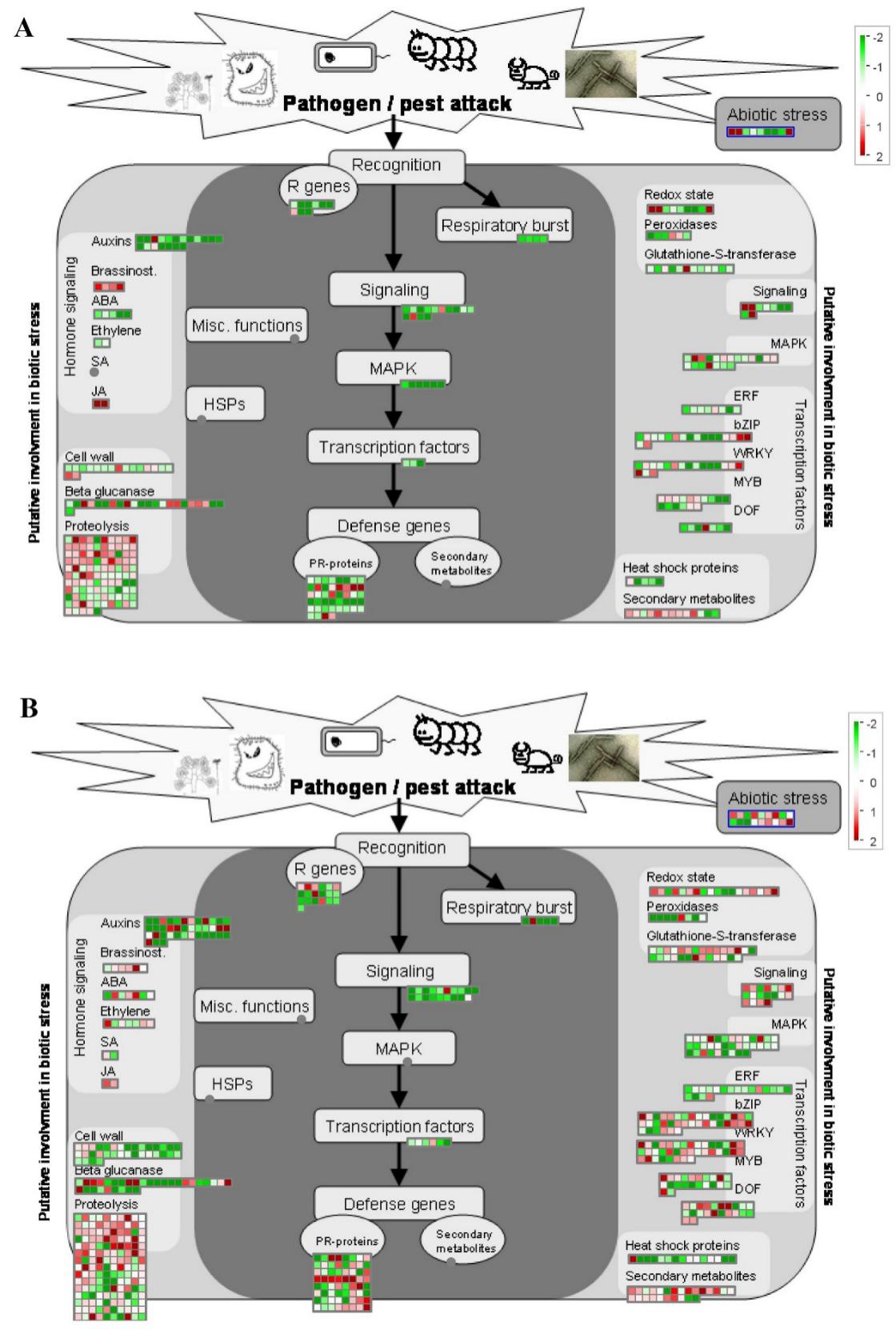


**Fig. S6**

Changes in DEGs expression during responses of plant to biotic stress in leaves (A) and buds (B) of ‘Kyoho’ grape after 5-azaC exposure. The changes of DEGs expression are appeared by the color of the grid, which green represents down regulation, while red represents up regulation.
